# Changes in Activity and Kinetic Properties of the Proteasome in Different Rat Organs during Development and Maturation

**DOI:** 10.1155/2010/230697

**Published:** 2010-03-31

**Authors:** A. Petersen, A. Honarvar, M. Zetterberg

**Affiliations:** ^1^Department of Medical Chemistry and Cell Biology, Institute of Biomedicine, The Sahlgren's Academy, The University of Gothenburg, Box 440, 405 30 Göteborg, Sweden; ^2^Institute of Neuroscience and Physiology/Ophthalmology, The Sahlgren's Academy, The University of Gothenburg, Sahlgren's University Hospital/Mölndal, 431 80 Mölndal, Sweden

## Abstract

The proteasome is considered the most important proteolytic system for removal of damaged proteins with aging. Using fluorogenic peptide substrates, the chymotrypsin-like, the trypsin-like, and the peptidylglutamyl peptidase activities of the proteasome were measured in the soluble fractions of liver, brain, and lens rat homogenates. Specific activity was significantly decreased in liver and brain homogenates with maturation of the animal, that is, from newborn (7 days old) to fertile rats (2–4 months old). Rat lens homogenate exhibited an increase in activity with maturation and also with aging. Chymotrypsin-like activity was stimulated by calcium and this proteolytic activity was significantly decreased with maturation of the rat brain. The Michaelis-Menten constant (*K*_*m*_) increased with age in rat liver and lens, indicating a loss of affinity for its substrates by the proteasome in the animal with maturation and aging. The present data suggest that the loss of function of the proteasome with maturation may be due to structural changes of the proteasome or a decreased content of regulatory components.

## 1. Introduction

In the science of aging, “the disposable soma theory”, introduced by Kirkwood in 1977 [1], has become a helpful concept in understanding of the processes that lead to senescence and death. According to this theory, the primary cause of aging is the accumulation of cellular and molecular damage, that arises because of evolved limitations in somatic maintenance and repair functions. One important form of such damage accumulation is seen in the build-up of aberrant proteins, which is reinforced by the age-related impairment of protein degradation [2].

There are two major proteolytical systems responsible for most intracellular protein turnover: the lysosomal and the ubiquitin-proteasome system. For reviews see Jung et al. 2009 [3] and Reinstein and Ciechanover 2006 [4]. Although protein degradation was originally considered as an unregulated event, the discovery of the ubiquitin-proteasome pathway in the early 1980s showed that this process is indeed highly regulated. Proteins destined for degradation by the proteasome are labeled with a small protein, ubiquitin (Ub), through a series of reactions involving three groups of enzymes: the Ub-activating enzyme (E1), the Ub-conjugating enzymes (E2s), and the Ub-ligases (E3s). Some of these conjugation-steps require ATP, but the proteasome can act through both ATP-dependent/-independent and Ub-dependent/independent mechanisms. In recent years, the proteasome has been implied in highly controlled events such as cell cycle regulation and processing of antigens for presentation by the MHC class I pathways [5]. However, it was first described as a protein quality control system, where misfolded or damaged proteins were preferentially degraded by the proteasome. The ubiquitin-proteasome pathway is therefore a potential key system in the biological defense against aging, but although several studies have been performed to study age-related changes to the proteasome itself, data have been inconclusive [6–12].

The purpose of the present study was to investigate changes in proteasomal activity in several organs, including the liver, the brain, and the eye lens, during the lifespan of rats.

## 2. Material and Methods

### 2.1. Animals

Female white Sprague Dawley rats, 2–12 months old, were purchased from Iffa Credo, France, and kept at the animal department at Göteborg University for a maximum of one year. For the youngest age groups in the study, new rats, male and female, were generated through breeding with male rats from Charles River Laboratories, Germany. The animals were treated in accordance with the NIH statement for the use of animals in research and was approved by the regional ethics committee on animal experimentation at the University of Gothenburg. The following age groups and number of rats were included in the study: 7 days (*n* = 23), 2 months (*n* = 6), 4 months (*n* = 6), 10 months (*n* = 4), 18 months (*n* = 3–5), and 24 months (*n* = 4). All animals were sacrificed by CO_2_-gas inhalation and the organs of interest (liver, brain, eyes) removed and stored at −152°C until use. Prior to freezing, the eyes were dissected through a posterior approach and the lenses were kept at −152°C. Only clear lenses and organs without obvious pathology (i.e., tumors) were used.

### 2.2. Preparation of Rat Organs

Prior to analysis, rat organs were thawed, weighed, and homogenized on ice in 20 mM Tris buffer (pH 7.5), containing 5 mM EDTA, 3 mM NaN_3_, and 1 mM DTT. Homogenization was performed in a teflon/glass homogenizer with 10 up-and-down strokes at 1,500 rpm yielding homogenates of about 10% (for liver and brain tissues) and 0.3% (lens homogenates) respectively. The samples were centrifuged at 100,000 g for 60 min at +4°C after which the supernatant was saved for proteolytic analysis. Protein concentration was determined using Pierce's BCA Protein Assay Reagent B (Pierce, Rockford, Illinois) and bovine serum albumin was used as standard.

### 2.3. Proteolytic Activity

The synthetic peptide substrates used, Suc-Leu-Leu-Val-Tyr-AMC (LLVY) and Boc-Val-Gly-Arg-AMC (VGR) were purchased from Bachem Feinchemikalien AG, Switzerland and Z-Leu-Leu-Glu-AMC (LLE) was from Sigma Chemical CO, St Louis, MO. Stock solutions of the three peptide substrates used in the study were prepared as follows: LLVY and LLE were diluted to 40 mM in 100% DMSO, whereas VGR was suspended to 10 mM in 6% DMSO/aq. Prior to the proteolytic assay, substrates were diluted in 20 mM Tris (pH 7.5), with 5 mM EDTA and 3 mM NaN_3_ and DTT was added to yield a final concentration of 2 mM in the assay. For determination of *K*_*m*_-values a wide range of substrate concentrations was used; for LLVY between 12.5 and 100 *μ*M, for VGR 0.125 to 1 mM, and for LLE the substrate concentration was varied between 62.5 and 500 *μ*M (liver) or between 15 and 125 *μ*M (brain and lens). For determination of specific activity, final substrate concentrations of 50 *μ*M (LLVY), 500 *μ*M (VGR), and 62.5 *μ*M (LLE) were used. Specificity of the assay was ensured using the proteasome specific inhibitor lactacystin (10 *μ*M final concentration). Proteolytic experiments were also performed with addition of 5 mM ATP and 0.02% SDS, respectively, using 50 *μ*M LLVY as substrate.

Since LLVY is also a substrate for the calcium-dependent protease calpain, additional experiments were run where proteolysis was measured with 50 *μ*M LLVY in the presence of 1 mM calcium (1 mM excess over EDTA). After subtraction with corresponding proteolytic activity measured without calcium, the obtained difference w/wo calcium was considered calcium-dependent. To ensure that the activity thus detected was due to calpain, the assay was run with addition of the specific calpain inhibitor calpastatin (10 *μ*M final concentration). In lens homogenates from older rats, addition of calcium had a slightly inhibitory effect in itself, thus yielding a negative net calcium-dependent activity according to the formula above. In these cases, activity was set to zero.

At the start of the proteolytic assay, 100 *μ*L of supernatant and 100 *μ*l of substrate solution were added to a 96-well plate (Dynex Technologies) and fluorescence of the cleavage product was measured over time at 37°C in a microplate spectrofluorometer (SPECTRAmax GEMINI, Molecular Devices, Sunnyvale, CA), excitation wavelength 380 nm, and emission 440 nm. Activity was normally measured during 2 hours and *V*_max _ determined using SOFTmax PRO Version 2.6 as software (Molecular Devices, Sunnyvale, CA). Proteolytic activity is expressed as relative fluorescence units per second and gram of protein (RFU × s^−1^× g^−1^) and *K*_*m*_-values were obtained from Lineweaver-Burk plots.

### 2.4. Statistical Analysis

Proteolytic activity was measured in triplicates for each sample. To determine if *K*_*m*_-values were affected by age, regression analysis was performed. For evaluation of changes in specific activity the rats were divided into the following age groups: newborn (7 days old, *n* = 23), young/fertile (2–4 months old, *n* = 12), middle aged (10 months, *n* = 4), and old (18–24 months old, *n* = 7–9), and statistical analysis was then performed using ANOVA with Bonferroni as post hoc test to compensate for multiple comparisons. A *P*-value of less than  .05 was considered statistically significant. As software, Statview (Abacus Concepts, Inc., Berkeley, CA) and SPSS 16.0 (SPSS, Inc., Chicago, Il) were used.

## 3. Results

Activity of the three proteasomal peptidases (chymotrypsin-like, trypsin-like, and peptidylglutamyl peptidase activities) varied between age groups as shown in Tables 1, 2, and 3. In rat brain, all three peptidases exhibited a significant decrease in specific activity from 7 days (newborn) to 2–4 months of age (young/fertile). A significant drop in proteasomal activity between these two age groups was also seen in rat liver, with regard to trypsin-like and peptidylglutamyl-peptidase activities; for the chymotrypsin-like activity, the decreased peptidase activity between 7 days to 2–4 months was nonsignificant. No significant changes in proteasome activity was seen in rat liver and brain after the age of 2–4 months for any peptidases, except for the chymotrypsin-like activity in liver, where a slight increase could be detected with aging. Proteasome activity in rat lens homogenate differed markedly from that of liver and brain. All three peptidases activities increased continuously with maturation and aging, from newborn to the very oldest animals. Addition of 5 mM ATP and 0.02% SDS, respectively, using 50 *μ*M LLVY as substrate, did not stimulate proteolytic activity (not shown).

Since LLVY can be a substrate for both the proteasome (chymotrypsin-like activity) and the calcium-activated protease calpain, the assay using LLVY was performed both without calcium (5 mM EDTA) and with calcium (1 mM excess of Ca^2+^). The ratio of mean calcium-dependent versus mean calcium-independent proteolysis of LLVY was 24% (liver), 33% (brain), and 13% (lens), respectively for the newborn group. For all organs investigated, there was a decrease in calcium-dependent LLVY degradation between the two youngest age groups (Tables 1–3), and in rat lens, calcium-dependent activity was nondetectable for the older age groups (Table 3). In most of the lenses from fertile and old rats, addition of calcium even had a slightly inhibitory effect on proteolysis of LLVY (not shown). To investigate the nature of the calcium-dependent degradation of LLVY, calpastatin, an endogenous inhibitor of calpain, was added to the assay. However, no inhibition by calpastatin was seen (not shown). In contrast, addition of the proteasome-specific inhibitor lactacystin reduced calcium-dependent proteolysis to 10% of control. Calcium-independent degradation of LLVY was inhibited by lactacystin to 2% as compared to without inhibitor (rat brain homogenate, data not shown).

**Figure 1:**
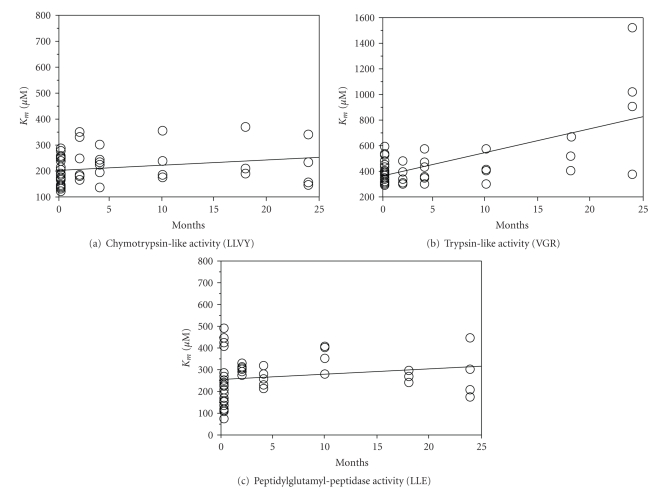
Michaelis-Menten constants (*K*_*m*_) of the proteasome in rat liver with age: regression plots of Michaelis-Menten constants (*K*_*m*_) of the proteasome in rat liver versus age. *K*_*m*_-constants were obtained from Lineweaver-Burk plots generated from proteolytic runs using four different substrate concentrations. For each concentration, triplicate samples were run. (a) Chymotrypsin-like activity: *R* ^2^ of the regression line is 0.049, *P* = .138. (b) Trypsin-like activity: *R* ^2^ = 0.417, *P* < .001. (c) Peptidylglutamyl peptidase activity: *R* ^2^ = 0.034, *P* = .219. A *P*-value of less than  .05 was considered statistically significant.

**Figure 2:**
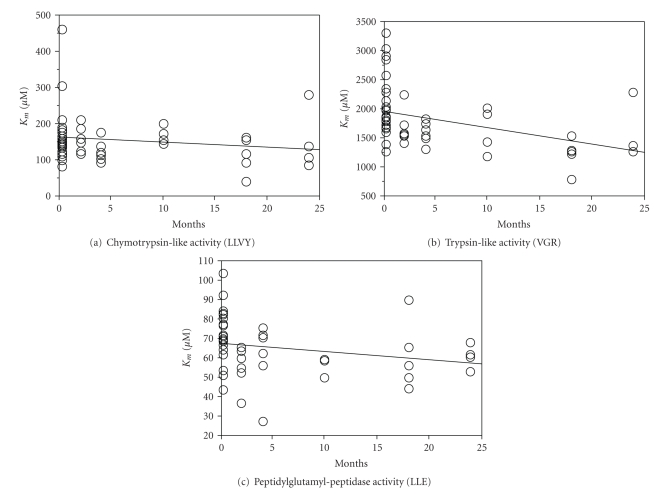
Michaelis-Menten constants (*K*_*m*_) of the proteasome in rat brain with age. Michaelis-Menten constants of the proteasome in rat brain plotted against age: *K*_*m*_-values were obtained as described in Figure 1. (a) Chymotrypsin-like activity: *R* ^2^ = 0.024, *P* = .291. (b) Trypsin-like activity: *R* ^2^ = 0.189, *P* = .002. (c) Peptidylglutamyl peptidase activity: *R* ^2^ = 0.060, *P* = .094. A *P*-value of less than  .05 was considered statistically significant.

**Figure 3:**
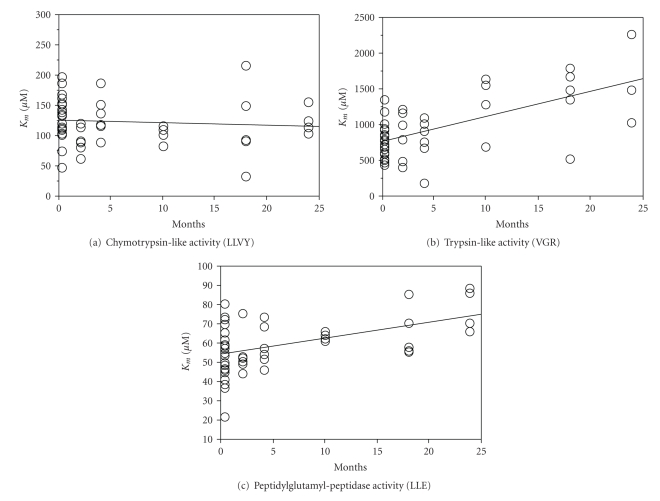
Michaelis-Menten constants (*K*_*m*_) of the proteasome in rat lens with age: Effect of aging on Michaelis-Menten constants of the proteasome in rat lens. *K*_*m*_-values were calculated as previously described (Materials and Methods, legend Figure 1). (a) Chymotrypsin-like activity: *R* ^2^ = 0.008, *P* = .535. (b) Trypsin-like activity: *R* ^2^ = 0.433, *P* < .0001. (c) Peptidylglutamyl peptidase activity: *R* ^2^ = 0.242, *P* = .0004. A *P*-value of less than  .05 was considered statistically significant.

Proteolytic activity was measured using different substrate concentrations and the resulting *V*_max _ was used for generation of Lineweaver-Burk plots, from which Michaelis-Menten constants (*K*_*m*_-values) were obtained. *K*_*m*_-values for all three main proteasomal peptidases were plotted against age and analysed using simple regression. In rat liver, all three peptidase activities exhibited increasing *K*_*m*_-values with maturation/aging, although only the increase in *K*_*m*_ for the trypsin-like activity was significant (Figure 1). In rat lens, *K*_*m*_ was significantly elevated with age for both the trypsin-like and the peptidylglutamyl peptidase activity, whereas the chymotrypsin-like activity exhibited a nonsignificant decline (Figure 3). Proteasome activity in rat brain differed from liver and lens by showing decreasing *K*_*m*_-values for all three peptidase activities with higher age (Figure 2). However, only the decline in trypsin-like activity was statistically significant.

## 4. Discussion

Although in recent years several studies have shown that the activity of the proteasome decreases with aging [6–9], contradictory data on this issue has long been and still is a fact [10–12]. In this study, the trypsin-like and the peptidylglutamyl peptidase activities in the soluble fraction of rat liver homogenate were significantly lower in rats older than 2 months of age as compared to newborn rats (7 days old). After the age of 2–4 months, no change in trypsin-like or peptidylglutamyl peptidase activities could be seen, indicating that the changes that occur in proteasome activity with age are more pronounced during the transition from sexually immature to mature animal than during senescence. In accordance with these data, a previous report by Conconi and Friguet on purified proteasome from rat liver reported that *V*_max _ of the chymotrypsin- and the trypsin-like activities was unaffected during aging, whereas the peptidylglutamyl peptidase activity exhibited an age-related decline [13].

In this study, the soluble fraction from brain homogenate of mature and older rat groups (from 2 months to 24 months) exhibited significantly lower activity of all three proteasomal peptidases as compared to the newborn group (7 days). Again, this may reflect changes in proteasomal function during sexual maturation of the animal, coinciding with a transition from an immature/newborn stage to a young adult/fertile stage. The ubiquitin-proteasome system has been ascribed a crucial role in neuronal protein turnover during development of the nervous system [14]. This decline in proteasome activities in rat brain is consistent with previous data from Zeng et al., demonstrating an overall decrease in chymotrypsin- and trypsin-like, as well as in peptidylglutamyl peptidase activity in middle aged (15 months) as compared to immature (6 weeks) rats [15]. Interestingly, they discovered differences between brain regions and it was only in substantia nigra that all three peptidase activities were significantly decreased, implying a role in Parkinson's disease.

The present data do not demonstrate any change in proteasomal activity in the aging rat brain after 2 months of age, consistent with a study from Abd El Mohsen et al., who did not see any effect by age in rat brain neither in the frontal cortex, hippocampus, nor in the cerebellum, when comparing rats of 3–5 months and 15-16 months of age [10]. Data are thus conflicting and the discrepancies between studies may reflect differences in species used or selected age groups. It may also be explained by different preparation/purification modes or techniques for proteolytic analysis. Most studies on the proteasome have been performed on the purified enzyme. However, it has been suggested that isolation and purification processes lead to activation of the enzyme [16] as well as to structural changes [17]; so characterization of age-related changes to the proteasome in this study was carried out in the soluble fraction of crude tissue homogenates under the assumption that it would give a physiologically more relevant assessment of proteasome activity. Both endogenous activating and inhibiting factors of the proteasome have been isolated [16, 18], indicating the importance of conditions as close to the native as possible when performing activity measurements.

Cataract is defined as an opacification of the eye lens leading to impaired vision. Although the exact mechanism for cataract development has not been elucidated, it is well known that with aging there is an increasing accumulation of posttranslationally modified proteins, which accumulate to form light-scattering high-molecular weight aggregates [19, 20]. The aged lens proteins exhibit oxidative changes and since the proteasome is considered the most important system in removal of oxidatively damaged proteins [21, 22], this proteolytic machinery has been suggested as an important mechanism to prevent cataract formation [23]. In the present study, no age-related decline in proteasome activity could be seen in rat lens homogenate. Instead there was a slight increase in all three peptidase activities with aging. It should be remembered, however, that proteolysis was measured in the soluble fraction of the lenses. Due to numerous posttranslational changes, there is increased hydrophobicity of the lens proteins with aging and a gradual redistribution of proteins from the soluble to the insoluble fraction. It is possible that there is also a parallel transition of the proteasome from the soluble to the insoluble proteins, since the latter should constitute more attractive substrates for the proteasome, containing a higher proportion of damaged proteins. This could be one explanation to the decrease in proteasome activity found in the soluble fraction of rat lenses in this study. Another possibility is of course an upregulation of proteasome activity with aging, as a result of higher demands for proteolytic degradation. Such an upregulation must, however, occur in the superficial parts of the lens, that is, the epithelium and the outermost lens fiber layers, since the interior part of the lens lacks cell nuclei as well as organelles.

The lack of proteolytic decline of the lens proteasome with aging is in accordance with previous work from our group, where no correlation of the chymotrypsin-like proteasomal activity with age could be seen in human lens epithelium [24] nor was there a decrease in any of the three proteasomal peptidase activities of human lens nuclei with aging [25]. However, proteasome activity was significantly lower in lens epithelium or lens nuclei from cataractous lenses/lens nuclei as compared to clear lenses/lens nuclei, but whether this is a primary cause or secondary effect is not clear [24, 25]. A report from Viteri et al. did demonstrate decreasing proteasome activity in human lenses with aging [26]. All three peptidase activities declined with aging, although the peptidylglutamyl peptidase activity was the most vulnerable, exhibiting a significant decrease already for 26–35-year-olds.

Degradation of the substrate LLVY was higher in the presence of 1 mM Ca^2+^ than without calcium (5 mM EDTA). Addition of the calpain-inhibitor calpastatin did not reduce calcium-stimulated activity, whereas lactacystin inhibited the major part of the calcium-dependent LLVY-degradation. This indicates that calpain activity was not present, or at least at a very low rate. Instead, calcium-dependent activity thus measured was mainly due to the chymotrypsin-like activity of the proteasome. It has been demonstrated that the proteasome may be regulated by calcium through binding to a 29 kDa activator subunit [27]. This is supported by findings from our group indicating that intracellular proteasome activity in human lens epithelium may be stimulated by calcium both from external and internal stores [28]. Previous findings from our group also demonstrate that the chymotrypsin-like activity of the proteasome in intact mouse lens can be stimulated by increased intracellular Ca^2+^-levels [29]. The present study is the first to demonstrate that calcium-stimulated chymotrypsin-like activity of the proteasome decreases with a transition from sexually immature (7 days old) to mature/fertile (2–4 months) animal. This decline was significant in the soluble fractions from rat brain homogenate as well as from lens homogenate. In rat lens, Ca^2+^-stimulated chymotrypsin-like activity was completely abolished in the older age groups and addition of calcium even had an inhibitory effect on proteasome activity. Regulation of the proteasome by fluctuations in intracellular calcium-levels provides an additional mode of regulation than the previously described regulatory mechanisms through ubiquitination, ATP-levels, GSH/GSSG-ratio, phosphorylation, regulatory subunits, and different proteasome-associated proteins [30–34], further emphasizing the importance for strict regulation of the proteasome system.

Most studies on age-related changes in proteasome activity have measured specific activity of purified proteasome or tissue homogenates. Few studies have investigated changes in kinetic properties such as *K*_*m*_ with aging. The Michaelis-Menten constant is defined as the substrate concentration at 1/2 *V*_max _. A high *K*_*m*_ thus indicates that a higher substrate concentration is needed to obtain the same rate of enzyme activity than an enzyme with a lower *K*_*m*_ would need. Correlating the *K*_*m*_-values for a protease with aging hence gives a view of the age-related change in affinity for a specific substrate. In this study, the trypsin-like peptidase activity in rat liver as well as the trypsin-like and the peptidylglutamyl peptidase activities in rat lens exhibited significant increases in *K*_*m*_-values with age, indicating declining affinity of the proteasome for its substrates with maturation and aging. The trypsin-like activity in rat brain showed an age-dependent decrease in Michaelis-Menten constant however. No significant change in *K*_*m*_-value was seen for the remaining peptidase activities in rat liver, brain, and lens. In a previous study, Conconi et al. found no age-related effect on the Michaelis-Menten constant for any of the peptidase activities when comparing purified proteasome from the liver of 8-month-old versus 24-month-old rats [13]. Studies on the aged rat retina [9] and rat liver [8] also failed to demonstrate any significant differences in *K*_*m*_ for the chymotrypsin-like, the trypsin-like, and the peptidylglutamyl peptidase activities with age. Data from the present and from previous studies are thus still inconclusive about age-related changes in the proteasome's affinity for its substrates.

The mechanism for the age-related decline in proteasome activity seen in some previous studies is not fully elucidated although several possibilities have been discussed and partially investigated. A few studies have reported decreased expression of the 20S proteasome with aging [9, 26], while others have reported reduced contents of regulatory subunits (PA700 and PA28) and constant or even increased levels of the immunoproteasome [8]. Post-translational modifications of the proteasome with aging have also been reported [26] as well as an age-related reduction in ubiquitin conjugation cascade mRNA [35]. In addition, there is a paradoxal relationship between the proteasome and oxidative stress, which may affect its activity upon aging. Although the proteasome is known to prefer mildly oxidated proteins to native as substrates [36, 37], it has also been shown that proteasome activity is inhibited by large protein aggregates, which are often the result of oxidative modifications [38].

The present study, demonstrating increasing *K*_*m*_-values with maturation and aging in rat liver and rat lens, indicates that structural changes to the proteasome, leading to lower affinity for its substrates, may contribute to the age-related changes in proteasome activity. Our results do not support a decline in proteasome activity with senescence, however, but rather emphasize the importance of the ubiquitin-proteasome system in development and differentiation early in life.

## Figures and Tables

**Table 1:** Proteasome activity in rat liver.

Type of activity	Age	Mean ± SD	*P*-value
(Substrate)		(RFU × s^−1^× g^−1^)	
Chymotrypsin-like	newborn	2.67 ± 1.33	
activity (LLVY)	young/fertile	1.83 ± 0.69	N.S.
	middle aged	1.94 ± 0.16	N.S.
	old	3.73 ± 0.98	.004*

Calcium-dependent	newborn	0.64 ± 1.37	
chymotrypsin-like activity	young/fertile	0.24 ± 0.29	N.S.
(LLVY w 1 mM Ca^2+^	middle aged	0.32 ± 0.24	N.S.
- LLVY wo Ca^2+^)	old	0.18 ± 0.18	N.S.

Trypsin-like activity	newborn	7.43 ± 2.17	
(VGR)	young/fertile	3.29 ± 1.07	.001^†^
	middle aged	3.47 ± 0.82	<.001^†^
	old	4.01 ± 0.75	<.001^†^

Peptidylglutamyl-	newborn	2.50 ± 0.64	
peptidase activity (LLE)	young/fertile	1.24 ± 0.46	<.001^†^
	middle aged	1.18 ± 0.21	<.001^†^
	old	1.22 ± 0.20	<.001^†^

Chymotrypsin-like, trypsin-like, and peptidylglutamyl-peptidase activities in the soluble fractions of rat liver homogenate from newborn (7 days, *n* = 23), young (2–4 months, *n* = 12), middle aged (10 months, *n* = 4), and old (18–24 months, *n* = 7) rats. Chymotrypsin-like activity was also measured with an excess of calcium (1 mM). Calcium-dependent chymotrypsin-like activity was defined as the net difference between proteolysis of LLVY with and without calcium, respectively. Proteolytic activity is expressed as relative fluorescence units (RFUs) per second and gram of protein. Mean ± SD (standard deviation) is shown. Statistical analysis was made using ANOVA with Bonferroni as post hoc. A *P*-value of less than  .05 was considered statistically significant. N.S. = Not Significant. *Significant difference compared to the young group and ^†^the newborn group.

**Table 2:** Proteasome activity in rat brain.

Type of activity	Age	Mean ± SD	*P*-value
(Substrate)		(RFU × s^−1^× g^−1^)	
Chymotrypsin-like	newborn	8.05 ± 1.47	
activity (LLVY)	young/fertile	2.17 ± 0.50	<.001^†^
	middle aged	2.39 ± 0.44	<.001^†^
	old	2.76 ± 0.41	<.001^†^

Calcium-dependent	newborn	2.68 ± 0.48	
chymotrypsin-like activity	young/fertile	1.11 ± 0.28	<.001^†^
(LLVY w 1 mM Ca^2+^	middle aged	1.18 ± 0.29	<.001^†^
- LLVY wo Ca^2+^)	old	1.05 ± 0.38	<.001^†^

Trypsin-like activity	newborn	5.02 ± 0.81	
(VGR)	young/fertile	1.57 ± 0.22	<.001^†^
	middle aged	1.63 ± 0.30	<.001^†^
	old	1.77 ± 0.27	<.001^†^

Peptidylglutamyl-	newborn	6.41 ± 1.34	
peptidase activity (LLE)	young/fertile	1.72 ± 0.31	<0.001^†^
	middle aged	1.91 ± 0.25	<.001^†^
	old	2.00 ± 0.39	<.001^†^

Chymotrypsin-like, trypsin-like, and peptidylglutamyl-peptidase activities in the soluble fractions of rat brain homogenate from newborn (7 days, *n* = 23), young (2–4 months, *n* = 12), middle aged (10 months, *n* = 4), and old (18–24 months, *n* = 9) rats. Calcium-dependent chymotrypsin-like activity was defined as the net difference between proteolysis of LLVY with and without calcium, respectively. Proteolytic activity is expressed as relative fluorescence units (RFUs) per second and gram of protein. Mean ± SD (standard deviation) is shown. Statistical analysis was made using ANOVA with Bonferroni as post-hoc. A *P*-value of less than  .05 was considered statistically significant. N.S. = Not Significant. ^†^denotes significant difference compared to the newborn group.

**Table 3:** Proteasome activity in rat lens.

Type of activity	Age	Mean ± SD	*P*-value
(Substrate)		(RFU × s^−1^× g^−1^)	
Chymotrypsin-like	newborn	0.23 ± 0.14	
activity (LLVY)	young/fertile	0.28 ± 0.05	N.S.
	middle aged	0.30 ± 0.04	N.S.
	old	0.36 ± 0.11	.026*

Calcium-dependent	newborn	0.03 ± 0.06	
chymotrypsin-like activity	young/fertile	<0.00	N.A.
(LLVY w 1 mM Ca^2+^	middle aged	<0.00	N.A.
- LLVY wo Ca^2+^)	old	<0.00	N.A.

Trypsin-like activity	newborn	0.15 ± 0.07	
(VGR)	young/fertile	0.20 ± 0.04	N.S.
	middle aged	0.24 ± 0.03	N.S.
	old	0.37 ± 0.10	<.001*^†^,.030^‡^

Peptidylglutamyl-	newborn	0.21 ± 0.08	
peptidase activity (LLE)	young/fertile	0.29 ± 0.06	.040*
	middle aged	0.35 ± 0.04	.013*
	old	0.40 ± 0.09	<.001*,.008^†^

Chymotrypsin-like, trypsin-like, and peptidylglutamyl-peptidase activities in the soluble fractions of rat lens homogenate from newborn (7 days, *n* = 23), young (2–4 months, *n* = 12), middle aged (10 months, *n* = 4), and old (18–24 months, *n* = 9) rats. Calcium-dependent chymotrypsin-like activity was defined as the net difference between proteolysis of LLVY with and without calcium, respectively. Proteolytic activity is expressed as relative fluorescence units (RFUs) per second and gram of protein. Mean ± SD (standard deviation) is shown. Statistical analysis was made using ANOVA with Bonferroni as post hoc. A *P*-value of less than  .05 was considered statistically significant. N.A. = Not Applicable; in these cases addition of calcium caused an inhibition of the activity, yielding a net negative calcium-dependent activity. N.S. = Not Significant. Significantly higher activity as compared to *newborn, ^†^young and ^‡^middle aged groups.
